# Investigation of the Distribution and Content of Acetylcholine, a Novel Functional Compound in Eggplant

**DOI:** 10.3390/foods10010081

**Published:** 2021-01-04

**Authors:** Wenhao Wang, Shohei Yamaguchi, Ayako Suzuki, Naomi Wagu, Masahiro Koyama, Akihiko Takahashi, Risa Takada, Koji Miyatake, Kozo Nakamura

**Affiliations:** 1 Department of Science and Technology, Graduate School of Medicine, Science and Technology, Shinshu University, 8304, Minamiminowa, Nagano 399-4598, Japan; 20hs502e@shinshu-u.ac.jp (W.W.); 19hs505d@shinshu-u.ac.jp (S.Y.); 2 Midorigaoka Junior High School, 426, Kega, Iida, Nagano 395-0813, Japan; suzuki.ayako@ed.iidanet.jp; 3 Department of Bioscience and Biotechnology, Faculty of Agriculture, Shinshu University, 8304, Minamiminowa, Nagano 399-4598, Japan; wagun@shinshu-u.ac.jp; 4 Wellnas Co., Ltd., Toranomon Masters Building 6F, 1-12-14, Toranomon, Minato-ku, Tokyo 105-0001, Japan; mkoyama32@wellnas.biz; 5 Kochi Agricultural Research Center, 1100 Hataeda, Nankoku, Kochi 783-0023, Japan; akihiko_takahashi@ken4.pref.kochi.lg.jp; 6 Saladcosmo. Co., Ltd., 1-15, Sendambayashi, Nakatsugawa, Gifu 509-9131, Japan; saladlab@saladcosmo.co.jp; 7 Institute of Vegetable and Floriculture Science, NARO, 360 Kusawa, Ano-cho, Tsu 514-2392, Mie, Japan; miya0424@affrc.go.jp; 8 Institute of Agriculture, Academic Assembly, Shinshu University, 8304, Minamiminowa, Nagano 399-4598, Japan

**Keywords:** choline ester, acetylcholine, eggplant, LC–MS/MS, choline, cooking, functional food, eggplant part

## Abstract

Eggplants are rich in acetylcholine (ACh), which can improve high blood pressure and negative psychological states. However, information on ACh content in individual parts of eggplant and the changes in ACh content during eggplant development is limited. Therefore, we investigated the ACh content in various parts of eggplant, namely, the leaf, root, bud, calyx, ovary, fruit, exocarp, mesocarp, partition, placenta, core, fruit base, fruit center, and fruit top in 26 eggplant varieties. Furthermore, the effect of heat treatment on ACh content was investigated. The ACh content significantly differed among the eggplant varieties. The difference between the varieties with the highest and lowest ACh content was 100-fold (Tosataka: 11 ± 0.61 mg/100 g fresh weight (FW) and Ryoma: 0.11 ± 0.046 mg/100 g FW, respectively). Eggplant fruit presented the highest ACh content (4.8 mg/100 g FW); it was three times higher than that in other parts combined (1.6 mg/100 g FW). The root contained the lowest ACh content among all parts. The ACh content increased with growth after flowering. The ACh content in the fruit 1.5 months after flowering was 400 times that in the ovary. ACh was uniformly distributed in eggplant flesh. Heat treatment did not cause ACh loss in eggplant. Thus, eggplant is an excellent raw material for functional foods.

## 1. Introduction

Eggplant (*Solanum melongena*) belongs to the family Solanaceae. According to data obtained in 2018, the annual harvest of eggplant is 1,864,556 ha, ranking 10th in vegetable production globally [1]. Eggplant has been cultivated in Asia for more than 1500 years. The earliest record of its use as a crop can be found in a Sanskrit document, which dates to the beginning of the Christian era [2,3,4], and it probably originated in India [5]. Acetylcholine (ACh) is present in almost all organisms, including eubacteria, protozoa, and eukaryotes, and it functions as a neurotransmitter in vertebrates [6,7]. A previous study reported that eggplants—rich in ACh—administered orally showed blood pressure-reducing effect in spontaneously hypertensive rats, by inhibiting sympathetic nerve activity through the M3 muscarinic ACh receptor [8]. Nishimura et al., fellow researchers [9], conducted a clinical study to determine whether eggplant intake lowered blood pressure. The daily intake of eggplant powder containing 2.3 mg of ACh was found to improve blood pressure and psychological states. Their study revealed a new application of eggplants, indicating their potential use as a functional food for relieving stress in individuals with normal to high blood pressure or grade 1 hypertension. A survey of the choline ester content in 19 fresh agricultural products revealed that the ACh content in eggplant was over 2900 times higher than that in other fresh agricultural products [10]. The World Health Organization recommends that adults should consume at least 400 g of fruits and vegetables daily [11]. The 18 studied agricultural products, except eggplant (average: 2.11 × 10^−3^ mg/100 g fresh weight (FW)), can only supplement 8.44 μg ACh/day, which is only 0.4% of the effective dosage. However, eggplant (6.12 mg/100 g FW) only needs 38 g to supply sufficient ACh for improving blood pressure and psychological states [10].

ACh is a major functional compound in eggplant. It is important to understand how the ACh content changes among varieties and parts of eggplant and how it changes during eggplant development and under heat treatment for promoting the practical application of eggplant. However, there is a lack of relevant data. Therefore, in this study, we investigated the ACh content in 26 eggplant varieties cultivated in Japan, compared to the ACh content in individual parts, and investigated the changes in the ACh content after flowering and heat treatment.

## 2. Materials and Methods

### 2.1. Chemicals

Ultrapure water with a specific resistance of 18.2 MΩ/cm—produced using an Arium 611 Ultrapure Water System (Sartorius Co., Goettingen, Germany)—was used in the experiments. Methanol (high-performance liquid chromatography [HPLC] grade), formic acid, 1 mol/L hydrochloric acid, and choline chloride were purchased from Nacalai Tesque, Inc. (Kyoto, Japan). Sodium dihydrogen phosphate and disodium hydrogen phosphate were purchased from Fujifilm Wako Pure Chemical Industries, Ltd. (Osaka, Japan). ACh chloride was purchased from Kanto Chemical Co., Inc. (Tokyo, Japan). 2-Aminoethyl-trimethylammonium pivaloylamide (EN) was synthesized in our laboratory [10].

### 2.2. Eggplant and Tomato Samples

The origin of the 26 eggplant varieties was as follows: SL Shisui, Mizunasu, Wase-daimaru, Shoya onaga, Kurowashi (a), Moginasu, Oserikawa, TNA-112, Senryo, Ryoma (a), Chikuyo, and Senryo No. 2 were harvested in a research farm of Takii & Co., Ltd. (Konan City, Shiga Prefecture, Japan; growth period: May to July 2017; summer to autumn; Japanese varieties). White bell, Black bell, Jade, White, Rosabianca, White clara, Purple clara, Thailand, and Florence purple were harvested by Fujita Seed Co., Ltd. (Sanda City, Hyogo Prefecture, Japan; growth period: May to July 2017; summer to autumn; non-Japanese varieties). Tosataka, Shintaro, Ryoma (b), Kurowashi (b), and Touchikonasu were harvested in Kochi Agricultural Research Center (Nankoku City, Kochi Prefecture, Japan; growth period: June 2017 to January 2018; winter to spring; greenhouse 12 °C; Japanese varieties). 

The following agricultural products were used to quantify the plant components and were planted by the Institute of Vegetable and Floriculture Science, National Agriculture and Food Research Organization [NARO] (Tsu City, Mie Prefecture, Japan) in October 2017. Eggplant (Senryo No. 2) leaves, roots, buds, calyxes, ovaries, and fruits (1 week, 2 weeks, and 1.5 months) after flowering were used. As a control, we used similarly cultivated tomato (Home Momotaro) leaves, roots, flowers, and fruits (2 weeks and 2 months) after flowering.

**Figure 1:**
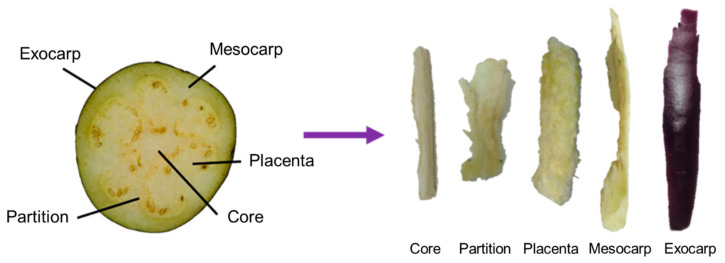
Fruit parts of eggplant (Tosataka).

Tosataka was harvested in January 2018 from Kochi Agricultural Research Center and was divided into exocarp, mesocarp, outside placenta, inside placenta, and core (Figure 1). In addition, the eggplant fruit was divided into three parts from the root to the bottom: the fruit base (i.e., near the root), fruit center (the middle section), and fruit top (the part away from the root).

### 2.3. Sample Preparation for Quantification

The following heat treatment strategy was employed for Tosataka: nine eggplants in the same growth environment and growth period were selected, three as control samples, three as microwave-heated samples, and three as fried-heat samples. The samples to be heat-treated were cut into 2–3 cm pieces, washed with water for 15 s, and then dehydrated for 10 s. After cleaning, the microwaved eggplant sample was heated at 700 W for 1.5 min/70 g (the inner temperature of the eggplant was approximately 96 °C), and this was freeze-dried into a powder for quantification. After frying the eggplant sample in oil at 180 °C for 2 min, the oil on the surface was wiped off using an absorbent paper, and the sample was then freeze-dried for quantification. The treatment of control samples and other samples is described below.

After washing the surface and wiping off the moisture, the edible portion was cut into 2–3 cm squares and the fresh weight was recorded; then, samples were lyophilized in a freeze dryer (FDU-2000; Tokyo Rikakikai Co., Ltd., Tokyo, Japan). The dry yield is shown in Tables S1–S4. The lyophilized sample was ground in a mill mixer (31,000 rpm, 1.5 min; MNN-2001; Tokyo Unicom, Tokyo, Japan) to a powder after recording the dry weight. The lyophilized powder (10 mg) was weighed into a 1.5-mL tube and the EN internal standard (10 μL) was added. Thereafter, 50 mmol/L HCl (190 μL) was added and mixed for 3 min by vortexing, followed by centrifugation (1000× *g*, 25 ± 5 °C, 3 min) to obtain the supernatant. Subsequently, 50 mmol/L HCl (200 μL) was added to the residue, and the steps stirring, centrifugation, and supernatant collection were repeated twice. All collected supernatants were combined (approximately 600 μL), and 1 mol/L phosphate buffer (PBS, 300 μL, pH = 7, 340.4 mmol/L sodium dihydrogen phosphate, and 659.6 mmol/L disodium hydrogen phosphate) was added as the extraction sample.

Next, a weak acid cation exchange column was used for solid-phase extraction of the extracted samples (InertSep CBA 100 mg/1 mL; GL Sciences, Tokyo, Japan). The solid-phase extraction column was activated with methanol (1.6 mL) and pure water (1.6 mL), and 10 mmol/L PBS (8 mL) was added to neutralize its pH; then, the extracted sample (approximately 900 μL) was added. The sample was stabilized with 10 mmol/L PBS (1350 μL), washed with pure water (2.4 mL), and eluted with 1 mol/L HCl (500 μL). The HCl-eluted solution was collected in a 1.0-mL volumetric flask, and then filled with mobile phase (33.0% [*v*/*v*] methanol containing 0.0100% formic acid) to obtain the pretreatment solution. Owing to the high ACh concentration in the eggplant sample, the pretreatment solution had to be diluted 200 times when quantifying ACh in the eggplant sample.

### 2.4. Quantification of ACh and Choline

The quantification of ACh and choline is based on a methodology described in previous studies [10,12]. An LC–MS/MS system (consisting of Nexera-i (ultra-performance liquid chromatograph) and LCMS–8045 (MS) from Shimadzu Co., Kyoto, Japan) was used. Chromatographic separation of ACh and choline was achieved using YMC-Triart PFP (4.6 mm × 250 mm, 5 μm) at 40 °C. Analytes were separated by elution with 33.0% (*v*/*v*) methanol containing 0.01% formic acid. A flow rate of 0.50 mL per min was employed; the injection volume was 1.0 μL and the analysis time was 25 min. The mass spectrometer was operated in the positive ionization mode (ESI [+] multiple reaction monitoring); the interface temperature and desolvation line (DL) temperature were 250 °C, the heat block temperature was 400 °C, the nebulizer gas flow was 3.0 L/min, and the drying and heating gas flow were 10 L/min. The following mass-to-charge ratio (*m/z*) transitions were monitored: 187.30 → 128.15 (EN); 146.15 → 87.10 (ACh); 104.20 → 45.05 (choline). These multiple reaction monitoring transitions were determined based on MS scan and product ion scan analyses. The voltage settings were as follows: Q1 Pre–Bias (V) was set to −13.0 (EN), −15.0 (ACh), and −11.0 (choline); collision energy (V) was set to −14.0 (EN), −15.0 (ACh), and −22.0 (choline); and Q3 Pre–Bias (V) was set to −24.0 (EN), −17.0 (ACh), and −17.0 (choline). ACh and choline were quantified using the standard addition method. The pretreatment solution was divided into three equal parts of 300 μL each. One part without standard solution was used as sample A. To the two remaining parts, a standard solution containing ACh and choline was added and used as samples B and C, respectively. A total of 600 μL was made for all three samples using the eluent (33.0% [*v*/*v*] methanol containing 0.0100% formic acid). The standard solution was added by assuming that the area value would be 1.5 times (sample B) or 2 times (sample C) that of sample A, based on analyses of sample A and a controlled reference standard solution. A calibration curve based on the area values from the MS/MS chromatograms was constructed. The concentration of ACh and choline was corrected based on the recovery of EN (internal standards). Finally, the content of ACh and choline in the lyophilized powder was converted to the content of ACh and choline per 100 g of fresh weight.

### 2.5. Statistical Analysis

All results are expressed as mean ± standard error. Tukey’s honestly significant difference test was performed when the analysis of variance revealed that the *p*-values for all results were < 0.05. Standard differences were considered significant at *p* < 0.05 when evaluated using Tukey’s honestly significant difference test (IBM SPSS statistics 24) and Student’s *t*-test (Microsoft Excel 2019 MSO [16.0.13328.20262]).

## 3. Results

### 3.1. Quantification of ACh in 26 Eggplant Varieties

**Figure 2:**
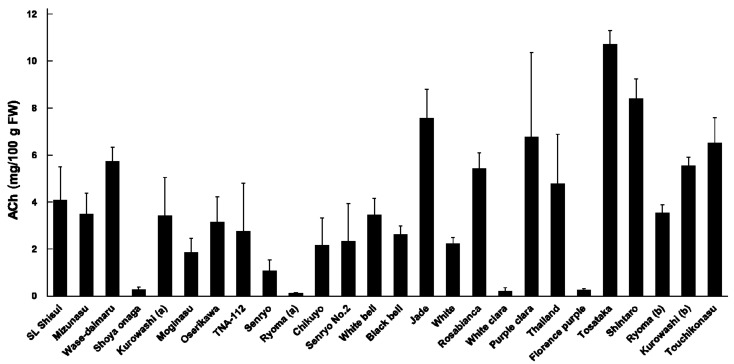
ACh content in 26 varieties of eggplant (*n* = 3). ACh: acetylcholine; FW: fresh weight. Kurowashi (a) and Ryoma (a) were harvested in summer; Kurowashi (b) and Ryoma (b) were harvested in winter.

A quantitative analysis of ACh in 26 eggplant varieties was performed by LC–MS/MS (*n* = 3, Figure 2). All eggplant varieties analyzed in this study contained ACh. In Japan, eggplant is cultivated throughout the year for commercial purposes. Therefore, we investigated the ACh content in eggplants harvested in different seasons to elucidate the seasonal changes in the ACh content. The average ACh content in all eggplants was 3.8 ± 0.53 mg/100 g FW, the maximum content was 11 ± 0.61 mg/100 g fresh weight (FW) (Tosataka), the minimum content was 0.11 ± 0.046 mg/100 g FW (Ryoma (a)), and the difference between the varieties with the maximum and minimum ACh content was 100-fold. The ACh content in Japanese eggplant varieties grown in summer to autumn was 0.11 (Ryoma (a)) to 5.7 (Wase-daimaru) mg/100 g FW, with an average of 2.5 ± 0.46 mg/100 g FW, whereas that in varieties originating outside Japan was 0.21 (White clara) to 7.6 (Jade) mg/100 g FW and the average was 3.7 ± 0.88 mg/100 g FW. The ACh content in Japanese eggplants grown in winter to spring was 3.5 (Ryoma (b)) to 11 (Tosataka) mg/100 g FW and the average was 6.9 ± 1.2 mg/100 g FW. The ACh content in eight parts of the eggplant was quantified. As a control, the ACh content in five parts of tomato (which belongs to Solanaceae) was quantified.

### 3.2. Quantification of ACh in Eggplant (Senryo No. 2) and Tomato (Home Momotaro)

A quantitative analysis of ACh in eight parts of eggplant and five parts of tomato was performed by LC–MS/MS (*n* = 3, Table 1). All eight parts of eggplant contained ACh. The part with the highest ACh content was the fruit, followed by the calyx, and the root contained the lowest ACh content. The ACh content in eggplant increased with growth after flowering. The ACh content in the fruit 1.5 months after flowering (4.8 ± 1.2 mg/100 g FW) was 400 times higher than that in the ovary (0.012 ± 0.0031 mg/100 g FW). Although both tomato and eggplant belong to the family Solanaceae, ACh could only be detected in trace amounts in tomato, or was not detected in certain parts; this was consistent with the findings of a previous study [10]. As the ACh content in eggplant fruit was the highest, the ACh content in six parts of eggplant fruit was subsequently analyzed.

### 3.3. Quantification of ACh and Choline in Six Parts of Eggplant Fruit (Tosataka)

A quantitative analysis of ACh and choline in six parts of eggplant fruit grown in the greenhouse at 12 °C in winter was performed by LC–MS/MS (*n* = 3, Table 2). The part with the highest ACh and choline content was the exocarp (ACh: 7.5 ± 0.20 mg/100 g FW; choline: 3.6 ± 0.21 mg/100 g FW). The lowest ACh content was observed in the partition (6.0 ± 0.54 mg/100 g FW) and the outer placenta (6.0 ± 0.29 mg/100 g FW); the lowest choline content was observed in the mesocarp (1.3 ± 0.24 mg/100 g FW). In the flesh, the highest ACh content was observed in the core (6.6 ± 0.26 mg/100 g FW), which was 1.1 times that in the partition (6.0 ± 0.54 mg/100 g FW) and outer placenta (6.0 ± 0.29 mg/100 g FW). Furthermore, there was no significant difference in the ACh content in various parts of the flesh.

Despite this, the difference between the highest and lowest ACh and choline content in the fruit was only 1.3- and 2.8-fold, respectively. The ACh content in each part of the eggplant fruit was almost the same. We then investigated whether gravity caused a difference in the distribution of ACh in the fruit.

### 3.4. Quantification of ACh and Choline in the Base, Center, and Top Parts of Eggplant (Tosataka)

A quantitative analysis of the ACh and choline content in the base, middle of the center, and top parts of the eggplant fruit from a plant grown in a greenhouse at 12 °C in winter was performed by LC–MS/MS (*n* = 3, Table 3). The ACh and choline content was not significantly different among the parts. The fruit base (7.0 ± 0.37 mg/100 g FW) contained the highest content of ACh, which was only 1.1 times higher than the lowest ACh content (in the fruit top; 6.2 ± 0.44 mg/100 g FW). The fruit base (2.4 ± 0.22 mg/100 g FW) and fruit center (2.4 ± 0.17 mg/100 g FW) contained the highest content of choline, which was only 0.10 mg/100 g FW higher than the lowest choline content (in the fruit top; 2.3 ± 0.27 mg/100 g FW). It can be concluded that gravity does not affect the distribution of ACh content in eggplant fruit.

### 3.5. Influence of Heat Treatment on ACh Content in Eggplant (Tosataka)

**Figure 3:**
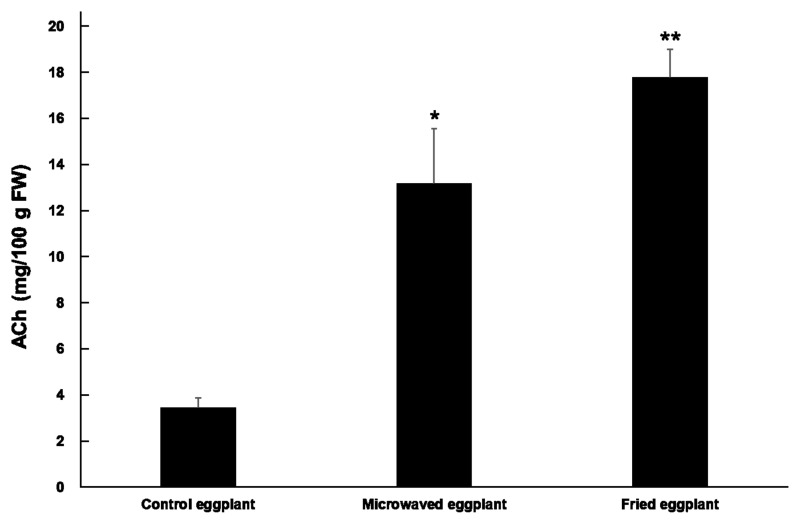
ACh content in the control, microwaved, and fried eggplant (*n* = 3). * *p* < 0.05, ** *p* < 0.01 versus the control eggplant, evaluated using Student′s *t*-test. ACh: acetylcholine; FW: fresh weight.

A quantitative analysis of ACh in uncooked (control), microwaved, and fried eggplant was performed by LC–MS/MS (*n* = 3, Figure 3). The ACh content in the control eggplant was 3.4 ± 0.44 mg/100 g FW, whereas that in the microwaved eggplant and fried eggplant was 13 ± 2.4 and 18 ± 1.2 mg/100 g FW, respectively. The ACh content in the microwaved and fried eggplants was higher than that in the control eggplant (*p* < 0.05 and *p* < 0.01, respectively). 

## 4. Discussion

In this study, we used LC–MS/MS to determine the content of ACh and/or choline in different varieties and different parts of eggplant. We also determined the changes in ACh and/or choline content in response to different heat treatments. To our knowledge, we are the first to conduct a comprehensive survey of ACh content in eggplant. ACh was identified in all 26 varieties of eggplant investigated. This was consistent with the findings of our previous study [10]. The ACh content was the highest in the eggplant fruit, and it was at the same level in each part of the fruit. The ACh content gradually increased with the growth of fruits and increased in response to heat treatments. 

The ACh content among the varieties was different, with a 100-fold difference between varieties with the highest and lowest contents. Based on the effective intake amount (2.3 mg of ACh/day) of eggplant required to improve the blood pressure and psychological states [9], we estimated that the required intake of varieties with the lowest and highest ACh content is 2.1 kg FW/day to only 21 g FW/day. The effective dose was different among the eggplant varieties. Therefore, we recommend that future studies should focus on identifying or cultivating eggplants with a high ACh content to reduce the effective daily intake and reduce the intake cost.

Here, the quantification of ACh in different parts of eggplant confirmed that all parts of eggplant contained ACh. The eggplant fruit (1.5 months after flowering) contained the highest content of ACh (4.8 mg/100 g FW), sometimes triple the sum of ACh present in the other parts (1.6 mg/100 g FW; leaf, root, bud, and calyx). The content of ACh in the fruit continued to increase with growth, and the content of ACh increased by 400-fold in just 1.5 months (fruit 1.5 months after flowering: 4.8 mg/100 g FW; ovary: 0.012 mg/100 g FW). Hence, we conclude that an extension of the harvest time is more beneficial for the accumulation of ACh in eggplants, resulting in increased food functionality. Almost no part of the tomato contains ACh, and therefore, the accumulation of ACh in the fruit was considered a characteristic of eggplants. In addition, we found that the ACh content in the same variety of eggplant cultivated in winter was generally higher than that cultivated in summer, and this may be related to the period from flowering to harvest.

Red pepper and eggplant belong to the family Solanaceae. Capsaicin, the pungent compound in red pepper, is only distributed in the partition and placenta of red pepper [13]. Therefore, in the process of extracting capsaicin, typically only these parts are used, which not only increases the cost of production but also generates waste. In contrast, ACh is uniformly distributed in eggplant flesh. Hence, there is no waste in the processing process, and the cost can be controlled. Therefore, eggplant is an excellent raw material for processing. In addition, all parts of eggplant fruit have considerable choline content, which further highlights the benefits of consuming eggplant.

Here, the ACh content in the eggplant was quantified after heat treatment. Cooking causes significant changes in the chemical composition of the raw material [14] and significantly increases the concentration of ACh in eggplant. This indicates the thermal stability of ACh and suggests an increase in the food functionality of eggplant. It has been reported that heat-treated eggplants exhibited a significantly higher total phenol and flavonol content and better antioxidant capacity than untreated eggplants [15,16,17]. Therefore, we recommend cooking eggplant, although it is not currently clear why the ACh content in eggplant increases after heating compared with that in unheated samples. When cut into pieces, the hydrolysis of ACh in eggplant cells upon contacting the extracellular acetylcholinesterase is a concern, because eggplant fruit has acetylcholinesterase [18,19,20]. Eggplant should be heated first, and then cut to retain its ACh content. In this study, we used only two heat treatments, microwave cooking and frying. In the future, we will further explore the influence of various heat treatments on the ACh content in eggplant.

## 5. Conclusions

The results of the present study showed that the ACh content varies among different varieties of eggplant, that the fruit of eggplant has the highest ACh content among the various eggplant parts, and that the ACh content increases with growth. Each part of the eggplant fruit presented the same level of ACh, and the ACh content was significantly higher in heat-treated eggplant than non-heated eggplant. In summary, the results of this study show the eggplant characteristics that make it suitable as a functional food material; eggplant varieties with a higher ACh content are undoubtedly excellent functional food materials. In this study, we focused on eggplants sold on the Japanese market. In the future, we will investigate the ACh content in eggplants around the world to identify varieties with the highest ACh content and provide the best raw materials for the relevant industries.

## Figures and Tables

**Table 1:** ACh content in eggplant (Senryo No. 2) and tomato (Home Momotaro) (*n* = 3).

Crop	Part	ACh (mg/100 g FW)
Eggplant (Senryo No. 2)	Leaf	2.5 × 10^−1^ ± 1.5 × 10^−1^
	Root	4.6 × 10^−3^ ± 2.1 × 10^−3^
	Bud	5.2 × 10^−1^ ± 3.7 × 10^−1^
	Calyx	8.2 × 10^−1^ ± 1.4 × 10^−2^
	Ovary (0-week fruit)	1.2 × 10^−2^ ± 3.1 × 10^−3^
	Fruit (1 week after flowering)	2.5 × 10^−1^ ± 7.7 × 10^−3^
	Fruit (2 weeks after flowering)	6.3 × 10^−1^ ± 1.8 × 10^−1^
	Fruit (1.5 months after flowering)	4.8 ± 1.2
Tomato (Home Momotaro)	Leaf	ND
	Root	ND
	Flower	ND
	Fruit (2 weeks after flowering)	4.2 × 10^−3^ ± 5.6 × 10^−4^
	Fruit (2 months after flowering)	ND

ACh: acetylcholine; FW: fresh weight; ND: not detected.

**Table 2:** ACh and choline content in six parts of the eggplant fruit (Tosataka) (*n* = 3).

Part	ACh (mg/100 g FW)	Choline (mg/100 g FW)
Exocarp	7.5 ± 2.0 × 10^−1^ ^a^	3.6 ± 2.1 × 10^−1^ ^a^
Mesocarp	6.6 ± 2.7 × 10^−1^ ^ab^	1.3 ± 2.4 × 10^−1^ ^b^
Partition	6.0 ± 5.4 × 10^−1^ ^b^	2.8 ± 7.2 × 10^−1^ ^ac^
Outer placenta	6.0 ± 2.9 × 10^−1^ ^b^	2.9 ± 4.4 × 10^−1^ ^ac^
Inner placenta	6.5 ± 1.9 × 10^−1^ ^ab^	1.9 ± 1.8 × 10^−1^ ^bc^
Core	6.6 ± 2.6 × 10^−1^ ^ab^	2.4 ± 5.1 × 10^−1^ ^abc^

Values within each column with different superscripts are different at *p* < 0.05, as evaluated using Tukey HSD. ACh: acetylcholine; FW: fresh weight.

**Table 3:** ACh and choline content in the base, center, and top of the eggplant fruit (Tosataka) (*n* = 3).

Part	ACh (mg/100 g FW)	Choline (mg/100 g FW)
Fruit base	7.0 ± 3.7 × 10^−1^	2.4 ± 2.2 × 10^−1^
Fruit center	6.7 ± 5.0 × 10^−1^	2.4 ± 1.7 × 10^−1^
Fruit top	6.2 ± 4.4 × 10^−1^	2.3 ± 2.7 × 10^−1^

No significant difference in values, as evaluated using an analysis of variance. ACh: acetylcholine; FW: fresh weight.

## Data Availability

All data, models, and code generated or used during the study are presented in the submitted article.

## Supplementary Materials

The following are available online at https://www.mdpi.com/2304-8158/10/1/81/s1, Table S1: Fresh weight, dry weight, and lyophilized yield of 26 varieties of eggplant (*n* = 3); Table S2: Fresh weight, dry weight, and lyophilized yield of eight parts of eggplant and five parts of tomato (*n* = 3); Table S3: Fresh weight, dry weight, and lyophilized yield of nine parts of the eggplant fruit (Tosataka) (*n* = 3); Table S4: Fresh weight, dry weight, and lyophilized yield of the control, microwaved, and fried eggplant (Tosataka) (*n* = 3); Figure S1: LC–MS/MS chromatogram of eggplant (representative data); retention time: 8.1 min (choline), 9.3 min (ACh), and 15.0 min (EN).
