# Correction: Wang et al. Investigation of the Distribution and Content of Acetylcholine, a Novel Functional Compound in Eggplant. *Foods* 2021, *10,* 81

**DOI:** 10.3390/foods11152298

**Published:** 2022-08-02

**Authors:** Wenhao Wang, Shohei Yamaguchi, Ayako Suzuki, Naomi Wagu, Masahiro Koyama, Akihiko Takahashi, Risa Takada, Koji Miyatake, Kozo Nakamura

**Affiliations:** 1 Department of Science and Technology, Graduate School of Medicine, Science and Technology, Shinshu University, 8304, Minamiminowa, Nagano 399-4598, Japan; 20hs502e@shinshu-u.ac.jp (W.W.); 19hs505d@shinshu-u.ac.jp (S.Y.); 2 Midorigaoka Junior High School, 426, Kega, Iida, Nagano 395-0813, Japan; suzuki.ayako@ed.iidanet.jp; 3 Department of Bioscience and Biotechnology, Faculty of Agriculture, Shinshu University, 8304, Minamiminowa, Nagano 399-4598, Japan; wagun@shinshu-u.ac.jp; 4 Wellnas Co., Ltd., Toranomon Masters Building 6F, 1-12-14, Toranomon, Minato-ku, Tokyo 105-0001, Japan; mkoyama32@wellnas.biz; 5 Kochi Agricultural Research Center, 1100 Hataeda, Nankoku, Kochi 783-0023, Japan; akihiko_takahashi@ken4.pref.kochi.lg.jp; 6 Saladcosmo. Co., Ltd., 1-15, Sendambayashi, Nakatsugawa, Gifu 509-9131, Japan; saladlab@saladcosmo.co.jp; 7 Institute of Vegetable and Floriculture Science, NARO, 360 Kusawa, Ano-cho, Tsu 514-2392, Mie, Japan; miya0424@affrc.go.jp; 8 Institute of Agriculture, Academic Assembly, Shinshu University, 8304, Minamiminowa, Nagano 399-4598, Japan

The authors wish to make a correction to the published version of their paper [1].

**Conflicts of Interest:** We declare that we have no financial and personal relationships with other people or organizations that can inappropriately influence our work, there is no professional or other personal interest of any nature or kind in any product, service and/or company that could be construed as influencing the position presented in, or the review of, the manuscript entitled, “Investigation of the Distribution and Content of Acetylcholine, a Novel Functional Compound in Eggplant”.

should be

**Conflicts of Interest:** M.K. is a shareholder-CEO in Wellnas Co., Ltd. K.N. is an inventor of pending (WO2015147251 and WO2018070545) and awarded (Japanese Patent No. 6192167 and Indonesia Patent No. P000063624) patents regarding this work and a shareholder in Wellnas Co., Ltd.

We apologize for this error. The change does not affect the scientific results. The published version will be updated on the article webpage, with a reference to this correction notice.
