# Supplementary material for: Investigation of the Distribution and Content of Acetylcholine, a Novel Functional Compound in Eggplant
Source: Foods. 2021 Jan 4;10(1):81. doi: 10.3390/foods10010081 (PMC7823263; doi:10.3390/foods10010081)
Supplement: Supplementary file 1 [file foods-10-00081-s001.pdf]

Table S1. Fresh weight, dry weight, and lyophilized yield of 26 varieties of eggplant (n = 3).

Cultivar	Fresh weight (g)	Dry weight (g)	Yield (%)
SL Shisui	55.96 ± 1.60	3.16 ± 0.10	5.65 ± 0.02
Mizunasu	55.23 ± 0.56	3.49 ± 0.10	6.32 ± 0.21
Wase-daimaru	62.43 ± 1.90	4.43 ± 0.31	7.11 ± 0.51
Shoya onaga	56.63 ± 1.30	3.84 ± 0.12	6.78 ± 0.22
Kurowashi (a)	63.63 ± 3.24	4.23 ± 0.33	6.62 ± 0.18
Moginasu	57.44 ± 0.39	3.44 ± 0.11	6.00 ± 0.22
Oserikawa	56.28 ± 0.76	3.40 ± 0.09	6.05 ± 0.17
TNA-112	54.40 ± 1.33	3.52 ± 0.27	6.46 ± 0.42
Senryo	56.79 ± 0.99	4.19 ± 0.09	7.39 ± 0.27
Ryoma (a)	56.15 ± 1.18	3.48 ± 0.09	6.19 ± 0.13
Chikuyo	55.49 ± 1.58	3.95 ± 0.18	7.11 ± 0.19
Senryo No. 2	58.64 ± 2.74	3.76 ± 0.10	6.44 ± 0.30
White bell	61.03 ± 2.28	15.20 ± 0.01	4.19 ± 0.09
Black bell	59.49 ± 1.28	15.19 ± 0.02	4.61 ± 0.11
Jade	61.31 ± 0.62	15.24 ± 0.02	4.66 ± 0.15
White	55.45 ± 2.05	15.28 ± 0.02	4.36 ± 0.05
Rosabianca	58.71 ± 2.48	15.23 ± 0.02	4.23 ± 0.22
White clara	53.74 ± 2.35	15.18 ± 0.02	4.82 ± 0.17
Purple clara	22.52 ± 2.70	15.18 ± 0.04	2.51 ± 0.34
Thailand	24.98 ± 1.84	15.15 ± 0.03	2.96 ± 0.23
Florence purple	59.82 ± 1.26	15.17 ± 0.03	5.27 ± 0.08
Tosataka	54.44 ± 1.36	3.68 ± 0.23	6.79 ± 0.59
Shintaro	55.72 ± 1.61	3.47 ± 0.03	6.23 ± 0.12
Ryoma (b)	56.12 ± 2.08	3.73 ± 0.13	6.66 ± 0.13
Kurowashi (b)	55.42 ± 1.20	3.63 ± 0.12	6.54 ± 0.12
Touchikonasu	53.38 ± 0.41	3.65 ± 0.02	6.83 ± 0.08

yield (%)—dry weight/fresh weight × 100.

Table S2. Fresh weight, dry weight, and lyophilized yield of eight parts of eggplant and five parts of tomato (*n* = 3).

Crop	Part	Fresh weight (g)	Dry weight (g)	Yield (%)
Eggplant (Senryo No. 2)	Leaf	4.83 ± 0.41	0.80 ± 0.07	16.56 ± 0.27
	Root	2.30 ± 0.08	0.29 ± 0.00	12.79 ± 0.32
	Bud	3.47 ± 0.38	0.66 ± 0.04	19.45 ± 0.95
	Calyx	3.30 ± 0.29	0.72 ± 0.03	21.94 ± 0.94
	Ovary (0-week fruit)	3.67 ± 0.52	0.44 ± 0.08	11.76 ± 1.67
	Fruit (1 week after flowering)	8.67 ± 0.34	0.80 ± 0.08	9.15 ± 0.60
	Fruit (2 weeks after flowering)	8.20 ± 0.22	0.69 ± 0.03	8.37 ± 0.15
	Fruit (1.5 months after flowering)	5.57 ± 0.89	0.52 ± 0.08	9.38 ± 0.29
Tomato (Home Momotaro)	Leaf	6.57 ± 0.80	1.09 ± 0.18	16.27 ± 1.10
	Root	2.43 ± 0.19	0.17 ± 0.02	6.96 ± 0.12
	Flower	5.63 ± 0.59	0.91 ± 0.09	16.51 ± 1.79
	Fruit (2 weeks after flowering)	4.00 ± 0.53	0.67 ± 0.03	17.42 ± 1.53
	Fruit (2 months after flowering)	7.23 ± 0.38	0.52 ± 0.03	7.13 ± 0.10

yield (%)—dry weight/fresh weight × 100.

Table S3. Fresh weight, dry weight, and lyophilized yield of nine parts of the eggplant fruit (Tosataka) (n = 3).

Part	Fresh weight (g)	Dry weight (g)	Yield (%)
Exocarp	1.35 ± 0.03	0.11 ± 0.00	8.16 ± 0.17
Mesocarp	22.35 ± 1.55	1.45 ± 0.11	6.49 ± 0.03
Partition	6.97 ± 0.58	0.57 ± 0.05	8.11 ± 0.01
Outer placenta	8.04 ± 1.28	0.63 ± 0.10	7.85 ± 0.02
Inner placenta	6.36 ± 0.09	0.46 ± 0.02	7.23 ± 0.23
Core	3.44 ± 1.48	0.25 ± 0.10	7.25 ± 0.15
Fruit base	19.58 ± 0.74	1.36 ± 0.03	6.95 ± 0.12
Fruit center	30.62 ± 1.43	2.26 ± 0.04	7.38 ± 0.22
Fruit top	25.60 ± 0.86	1.86 ± 0.07	7.24 ± 0.05

yield (%)—dry weight/fresh weight × 100.

Table S4. Fresh weight, dry weight, and lyophilized yield of the control, microwaved, and fried eggplant (Tosataka) (n = 3).

	**Fresh weight (g)**	**Dry weight (g)**	**Yield (%)**
Control eggplant	14.21 ± 0.36	0.86 ± 0.01	6.08 ± 0.20
Microwaved eggplant	10.15 ± 1.31	0.82 ± 0.09	8.09 ± 0.34
Fried eggplant	8.16 ± 1.01	1.61 ± 0.26	19.61 ± 0.84

yield (%)—dry weight/fresh weight × 100.

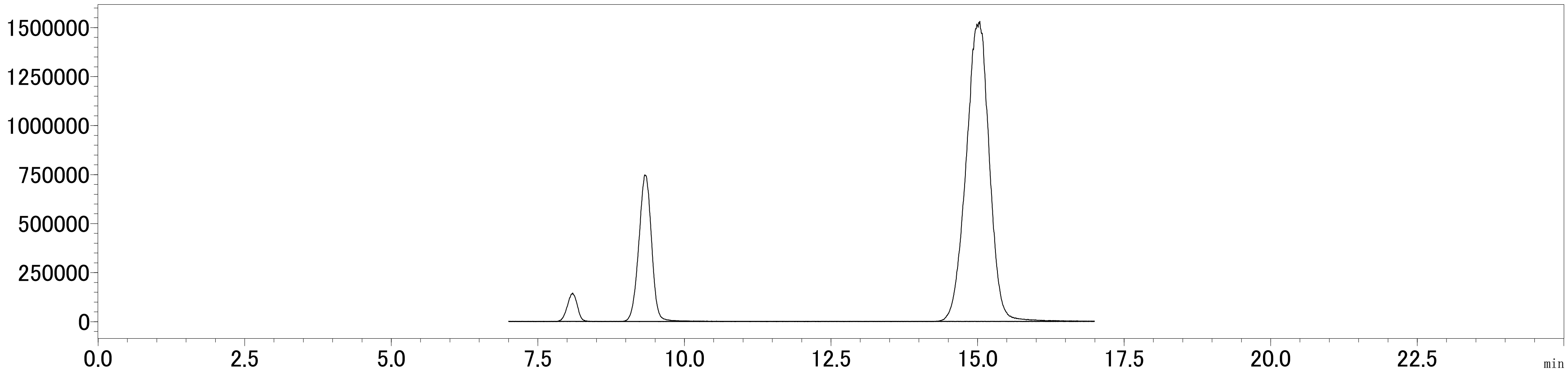

Figure S1. LC–MS/MS chromatogram of eggplant (representative data); retention time: 8.1 min (choline), 9.3 min (ACh), and 15.0 min (EN).
